# Descriptor-Guided Selection of Extracellular Vesicle Loading Strategies for Small-Molecule Drug Delivery: A Mechanistically Interpretable Decision-Support Framework

**DOI:** 10.3390/pharmaceutics18030384

**Published:** 2026-03-20

**Authors:** Romána Zelkó, Adrienn Kazsoki

**Affiliations:** University Pharmacy Department of Pharmacy Administration, Semmelweis University, Hőgyes Endre Street 7-9, 1092 Budapest, Hungary; kazsoki.adrienn@semmelweis.hu

**Keywords:** extracellular vesicles, drug loading strategies, elastic Net regression, molecular descriptors, decision-support modeling, formulation optimization, ranking-based prediction, nanocarrier systems

## Abstract

**Background:** Extracellular vesicles (EVs) are increasingly explored as nanocarriers in drug delivery; however, selecting an appropriate loading strategy for a given small-molecule cargo still relies largely on empirical, resource-intensive parallel screening within EV formulation workflows. Despite the widespread application of passive incubation, electroporation, saponin-mediated permeabilization, freeze–thaw cycling, and sonication, there is currently no mechanistically grounded, descriptor-informed framework that enables rational prioritization of loading methods during the early design stage of EV-based dosage forms, leading to inefficient trial-and-error experimentation. **Methods:** We assembled a chemically diverse dataset of 21 compounds with experimentally determined loading efficiencies across five EV loading methods and calculated seven mechanistically motivated physicochemical descriptors (LogP, molecular weight, aqueous solubility, hydrogen bond donors/acceptors, polar surface area, and formal charge) for each drug. Separate Elastic Net regression models were trained for each loading strategy. Model performance was evaluated using leave-one-out cross-validation, a predefined external validation set (*n* = 4), and 50 repeated random train–test splits. The analysis emphasized decision-level ranking of loading methods rather than the precise prediction of absolute efficiencies. The applicability domain was assessed via leverage analysis to define the supported chemical space for prospective implementation in EV-based formulation development. **Results:** As anticipated for biologically heterogeneous EV systems, continuous regression performance remained modest (LOOCV R^2^ = 0.06–0.41). In contrast, decision-level accuracy for identifying the experimentally optimal loading method was consistently high across validation schemes (internal: 76.5%; predefined external: 75%; repeated random validation: 80.5 ± 16.8%). Mechanical disruption methods (freeze–thaw and sonication) demonstrated comparatively greater predictive stability, while misclassification patterns suggested potential nonlinear behavior for highly polar, ionizable cargos. All compounds resided within the leverage-defined applicability domain, confirming adequate descriptor-space representation. **Conclusions:** This study establishes a mechanistically interpretable, descriptor-based decision-support framework capable of reliably prioritizing EV loading strategies for small-molecule cargos beyond empirical chance without altering standard protocols. By reframing the modeling objective from high-precision efficiency prediction to robust ranking of candidate methods, the approach offers a practical tool to triage between commonly used techniques, thereby reducing experimental burden in early-stage EV formulation development. The framework provides a quantitative basis for integrating molecular-descriptor-guided method selection into rational EV-based drug delivery design and can be expanded with membrane-specific descriptors and larger datasets.

## 1. Introduction

Extracellular vesicles (EVs) have emerged as biologically derived nanocarriers with increasing translational relevance in drug delivery including clinically oriented EV-based dosage forms now moving toward translation and first-in-human studies [1,2,3,4]. Their endogenous lipid bilayer structure, intrinsic biocompatibility, and ability to mediate intercellular communication render them promising systems for the transport of small molecules, nucleic acids, and biologics. While significant progress has been achieved in EV isolation, purification, characterization, and therapeutic exploration, rational formulation design remains a central challenge in the field [3,4,5].

Among the most critical unresolved formulation questions is the selection of an appropriate drug loading strategy. Multiple EV loading techniques are routinely employed, including passive incubation, electroporation, saponin-mediated permeabilization, freeze–thaw cycling, and sonication [6,7,8,9,10]. These approaches rely on fundamentally different physicochemical mechanisms. Passive incubation is primarily governed by diffusion and membrane partitioning processes. Electroporation induces transient aqueous nanopores via external electric fields [11]. Saponin interacts with membrane cholesterol, transiently increasing permeability [12]. Freeze–thaw cycling causes membrane phase transitions and structural rearrangement, whereas sonication introduces mechanical membrane disruption through shear forces [6,7].

Despite these mechanistic differences, loading strategy selection is commonly empirical [7,9,10]. In practice, multiple methods are frequently screened in parallel for each new compound, resulting in increased experimental burden, variability, and resource consumption. Notably, loading efficiency varies substantially between compounds and methods, suggesting that drug-specific molecular properties strongly influence loading behavior [9,10].

Drug–membrane interactions are governed by physicochemical descriptors such as lipophilicity, polarity, hydrogen bonding capacity, molecular size, solubility, and charge [13,14,15]. These parameters influence membrane partitioning, electrostatic interactions, pore-mediated transport, and permeabilization dynamics [13]. Descriptor-based modeling approaches, particularly quantitative structure–property relationship (QSPR) frameworks, have been widely applied in pharmaceutical sciences to predict solubility, permeability, and nanoparticle interactions [16,17]. However, to date, no systematic descriptor-informed decision framework has been proposed to rationally prioritize EV loading strategies.

Importantly, the complexity of EV systems presents inherent challenges for quantitative prediction. EV lipid composition varies depending on cellular origin and isolation method [1,2,3]. Membrane protein content, vesicle size distribution, and experimental loading conditions introduce additional variability. Under such conditions, insisting on high-R^2^ regression models may be statistically unrealistic and conceptually misaligned with the needs of formulation scientists. A more pragmatic and statistically tractable objective is decision-level prioritization: identifying, for a given compound, which loading strategy is most likely to perform best among a set of routinely available methods.

The present study therefore aimed to develop a molecular descriptor-informed, mechanistically interpretable decision-support framework for rational prioritization of EV loading strategies for small-molecule drugs, with the specific goal of streamlining early-stage EV formulation workflows and reducing the number of loading experiments required per candidate cargo.

To this end, twenty-one structurally diverse small-molecule compounds with experimentally determined loading efficiencies across five EV loading methods were analyzed. Seven physicochemical descriptors were incorporated into Elastic Net regression models to balance interpretability and regularization under limited sample size conditions. Model performance was evaluated using internal leave-one-out cross-validation, predefined external validation, and repeated random resampling to assess robustness. Furthermore, applicability domain analysis was incorporated to explicitly quantify descriptor-space coverage and extrapolation risk.

Because the available dataset is chemically heterogeneous but numerically limited, a regularized regression framework was required to balance interpretability with statistical stability. Elastic Net was selected as it combines L1 and L2 regularization, allowing shrinkage of correlated descriptors while retaining mechanistically meaningful contributions—an important consideration for formulation-oriented modeling where explanatory insight is prioritized alongside predictive utility.

By integrating mechanistic interpretability, external validation, robustness testing, and applicability domain assessment, this work proposes a structured, formulation-oriented decision-support framework for EV loading strategy selection. The findings provide proof-of-concept evidence that molecular descriptors can rationally inform EV loading prioritization and may reduce empirical screening burden in early-stage formulation development. In this study, the term “small-molecule cargo” refers to low-molecular-weight organic compounds (generally below approximately 1000 Da) and does not include peptides, proteins, or nucleic acids, whose loading mechanisms differ substantially and fall outside the scope of the present descriptor-based framework.

## 2. Materials and Methods

### 2.1. Experimental Dataset

The dataset consisted of twenty-one structurally diverse small-molecule drugs for which experimentally determined loading efficiencies (%) were available across five extracellular vesicle (EV) loading techniques:

1. Passive incubation;
2. Electroporation;
3. Saponin-mediated permeabilization;
4. Freeze–thaw cycling;
5. Sonication.

Loading efficiency (%) was defined as the percentage of drug incorporated into EVs relative to the total amount applied during the loading procedure.

The compounds were selected to represent broad physicochemical diversity in terms of lipophilicity, molecular weight, polarity, hydrogen bonding capacity, solubility, and charge state. This diversity was considered essential for evaluating descriptor-driven differences in loading behavior [18].

For each compound, the experimentally optimal loading method was defined as the technique yielding the highest measured loading efficiency.

The full experimental dataset is provided in Table 1 (Experimental Dataset), including molecular descriptors and loading efficiencies for all five methods.

### 2.2. Molecular Descriptors

Seven physicochemical descriptors were selected based on mechanistic relevance to membrane interaction processes:

- LogP (octanol–water partition coefficient);
- Molecular weight (MW);
- Aqueous solubility;
- Hydrogen bond donors (HBDs);
- Hydrogen bond acceptors (HBAs);
- Polar surface area (PSA);
- Formal charge.

These descriptors were chosen to represent key determinants of membrane partitioning, pore-mediated transport, electrostatic interaction, and permeabilization sensitivity.

Lipophilicity (LogP) influences membrane affinity and passive diffusion. PSA, HBD, and HBA characterize polarity and hydrogen bonding potential. Molecular weight impacts steric accessibility and diffusion kinetics. Solubility reflects aqueous compatibility and partition balance. Formal charge influences electrostatic interaction and electroporation behavior.

### 2.3. Data Preprocessing

#### 2.3.1. Missing Data Handling

Missing descriptor values were imputed using median imputation calculated from the training dataset.

#### 2.3.2. Standardization

Descriptors were standardized using z-score normalization:

(1) $$z_{j}=\frac{x_{j}-\mu_{j}}{\sigma_{j}}$$

where

- $x_{j}$ = original descriptor value;
- $\mu_{j}$ = mean of descriptor j;
- $\sigma_{j}$ = standard deviation of descriptor j;

Standardization ensures that coefficients reflect relative importance and prevents scale dominance in regularized regression.

Importantly, standardization parameters were calculated using the training dataset and applied consistently during validation to prevent data leakage.

### 2.4. Model Architecture

Separate regression models were constructed for each loading method.

For loading method m, predicted loading efficiency was defined as:

(2) $${\hat{y}}_{m}=\beta_{0,m}+\sum_{j=1}^{7}\beta_{j,m}z_{j}$$

where

- $\beta_{0,m}$ = intercept;
- $\beta_{j,m}$ = standardized regression coefficient;
- $z_{j}$ = standardized descriptor.

### 2.5. Subsection Regularization Strategy: Elastic Net

Given the limited dataset size (n = 21) and potential descriptor collinearity, Elastic Net regularization was employed.

The objective function minimized:

(3) $$\underset{\beta }{min}\left\{\frac{1}{n}\sum_{i=1}^{n}{\left(y_{i}-\beta_{0}-X_{i}\beta \right)}^{2}+\lambda [\alpha ||\beta |{|}_{1}+(1-\alpha )||\beta |{|}_{2}^{2}]\right\}$$

Elastic Net was selected because:

- L1 penalty (LASSO component) enables feature shrinkage and potential sparsity.
- L2 penalty (Ridge component) stabilizes coefficient estimation under multicollinearity.
- Combined penalty reduces variance while preserving interpretability.

Hyperparameters:

- α ∈ [0.1, 0.3, 0.5, 0.7, 0.9].
- λ selected via cross-validation over logarithmic grid.

Hyperparameter optimization was performed using Leave-One-Out Cross-Validation (LOOCV) on the training dataset.

All statistical analyses were performed in Python (version 3.12.1) using the scikit-learn library (version 1.8.0) for Elastic Net implementation. Data preprocessing, standardization, and model evaluation workflows were conducted using NumPy (version 2.4.2) and pandas (version 3.0.1). Hyperparameter tuning was performed via grid search within the Leave-One-Out Cross-Validation (LOOCV) framework. A fixed random seed was applied to ensure computational reproducibility.

### 2.6. Validation Process

#### 2.6.1. Internal Validation

Internal validation was conducted using LOOCV on the 17-compound training dataset. LOOCV was preferred over K-fold schemes due to the limited sample size (n = 21). In small datasets, K-fold partitioning further reduces already constrained training subsets and may introduce partition-dependent variance. LOOCV maximizes data usage in each iteration and provides a lower-bias estimate of generalization performance, which is appropriate for exploratory descriptor-based modeling aimed at method ranking rather than high-precision prediction.

In contrast to typical machine learning settings focused on maximizing predictive performance in large datasets, the present study operates in a small-n, mechanism-oriented modeling context where preserving descriptor–response relationships is critical. Under such conditions, LOOCV offers a more favorable bias–variance trade-off by avoiding additional variability introduced by repeated random partitioning. Therefore, LOOCV was considered more appropriate than K-fold validation for this exploratory, formulation-guided framework.

In each LOOCV iteration, one compound was held out while the models were trained on the remaining 16 compounds, after which predictions were generated for the excluded compound. Model performance was evaluated using Mean Absolute Error (MAE), Root Mean Square Error (RMSE), and the coefficient of determination (R^2^). Decision-level accuracy was calculated as the proportion of correctly predicted optimal loading methods.

#### 2.6.2. Predefined External Validation

Four compounds were predefined as external validation set:

- Sildenafil.
- Caffeine.
- Ampicillin.
- Furosemide.

These compounds were selected to represent broad physicochemical variability and approximate coverage of BCS classes, while BCS classification was not directly incorporated into modeling.

Models were trained on the remaining 17 compounds and applied to the external set.

Decision accuracy was calculated analogously.

#### 2.6.3. Repeated Random External Validation

Four compounds were randomly assigned to the external validation set, while the remaining seventeen compounds were used for model training. Elastic Net models were retrained for each split, and decision-level accuracy was calculated. The mean and standard deviation of accuracy across all repetitions were then reported to characterize model stability.

### 2.7. Validation Strategy

For each compound, predicted loading efficiencies were calculated for all five methods. The recommended loading strategy was defined as:

(4) $$\hat{m}=arg\;\underset{m}{max}\;{\hat{y}}_{m}$$

This ranking-based decision criterion aligns with formulation-relevant prioritization objectives.

### 2.8. Applicability Domain Assessment

To quantify descriptor-space coverage and extrapolation risk, leverage values were calculated using the hat matrix:

(5) $$H=X(X^{T}X{)}^{-1}X^{T}$$

Leverage threshold:

(6) $$h^{*}=\frac{3(p+1)}{n}$$

where

- p=7 descriptors;
- n=21 compounds.

Compounds with leverage values exceeding $h^{*}$ were considered outside the model’s applicability domain.

In prospective application, predictions for compounds exceeding this threshold should be interpreted with caution.

## 3. Results

### 3.1. Descriptor Space and Dataset Characteristics

The dataset presented in Table 1 encompasses compounds spanning a broad physicochemical spectrum, including substantial variability in lipophilicity, polarity, molecular size, hydrogen bonding capacity, solubility, and formal charge. This diversity enables systematic comparison of how chemically distinct cargos interact with mechanistically different EV loading approaches. By covering contrasting descriptor combinations, the dataset allows interpretation of loading behavior across diffusion-driven, permeabilization-driven, and mechanically induced incorporation mechanisms, thereby serving as an empirical foundation for formulation-relevant decision patterns rather than merely a descriptive compilation of efficiencies.

The 21-compound dataset covered broad physicochemical diversity. LogP values spanned from highly hydrophilic to moderately lipophilic molecules. Molecular weight distribution covered low- to medium-mass small molecules. Polarity-related descriptors (PSA, HBD, HBA) varied substantially, enabling evaluation of membrane partitioning versus permeabilization sensitivity. Formal charge distribution included neutral, positively charged, and negatively charged compounds.

This descriptor diversity was essential to ensure meaningful variability in loading behavior across mechanistically distinct EV loading methods.

The complete experimental dataset, including molecular descriptors and loading efficiencies across all five methods, is presented in Table 1.

### 3.2. Internal Cross-Validation Performance

Separate Elastic Net models were trained for each loading method using the 17-compound training set. LOOCV was employed to maximize statistical efficiency under limited sample size conditions.

#### 3.2.1. Continuous Regression Performance

Table 2 summarizes LOOCV regression metrics.

Given the inherent biological variability of EV systems and limited dataset size, moderate regression performance was anticipated.

#### 3.2.2. Decision-Level Performance

Despite moderate R^2^ values, decision-level performance was substantially stronger.

LOOCV decision accuracy reached 76.5% (13/17 correctly classified).

This indicates that descriptor information more robustly captures relative ranking of loading strategies than precise efficiency magnitude.

This distinction between regression precision and ranking robustness is critical from a formulation decision perspective.

### 3.3. Predefined External Validation

External validation was performed using four predefined compounds representing broad descriptor diversity (Table 3).

External decision accuracy was 75% (3/4).

The single misclassification (Ampicillin) is further examined in the Discussion section.

Importantly, external performance closely matched internal decision accuracy, suggesting limited overfitting.

### 3.4. Robustness Analysis: Repeated Random Validation

To assess generalization stability beyond a single predefined split, 50 repeated random external partitions were performed.

Across these iterations:

- Mean decision accuracy: 80.5%.
- Standard deviation: ±16.8%.

Decision accuracy ranged between 50% and 100%, reflecting expected statistical fluctuation under small sample conditions.

The robustness distribution indicates that model performance is not dependent on a single favorable partition but remains stable across resampling scenarios.

This strengthens confidence in the decision-support capacity of the framework.

### 3.5. Applicability Domain and Leverage Analysis

Leverage analysis yielded the following threshold: h* = 1.143.

All compounds within the dataset exhibited leverage values below this threshold, indicating adequate descriptor-space coverage.

This suggests that the training dataset provides sufficient chemical diversity within the explored descriptor space.

The applicability domain threshold enables prospective identification of extrapolation risk for new compounds.

### 3.6. Final Regression Equations

After validation, final deployable models were refitted using the full dataset (n = 21).

Passive Incubation:

(7) $${\hat{LE}}_{passive}=60.33+7.74z_{LogP}+4.56z_{MW}+3.47z_{Solubility}-2.81z_{HBD}-3.29z_{HBA}-2.83z_{PSA}+6.32z_{Charge}$$

Electroporation:

(8) $${\hat{LE}}_{electro}=12.79-4.81z_{LogP}-2.27z_{MW}-1.98z_{Solubility}+2.80z_{HBD}+2.25z_{HBA}+2.44z_{PSA}-4.34z_{Charge}$$

Saponin:

(9) $${\hat{LE}}_{saponin}=27.90-2.51z_{LogP}-1.01z_{MW}-2.49z_{Solubility}+3.46z_{HBD}+3.09z_{HBA}+2.80z_{PSA}-5.27z_{Charge}$$

Freeze–Thaw:

(10) $${\hat{LE}}_{freeze}=17.67-5.32z_{LogP}-0.93z_{Solubility}+4.63z_{PSA}$$

Sonication:

(11) $${\hat{LE}}_{sonic}=16.62-2.88z_{LogP}-2.55z_{MW}-4.19z_{Solubility}+3.26z_{HBD}+2.27z_{HBA}+3.26z_{PSA}-3.64z_{Charge}$$

These equations constitute the mathematical core of the decision-support framework.

For practical application, the final regression equations were implemented in an Excel-based decision-support tool provided in the Supplementary Materials (Table S1), which enables users to input compound descriptors and obtain the recommended EV loading method.

## 4. Discussion

### 4.1. Decision-Support Framing in a Biologically Heterogeneous EV System

The primary objective of the present study was not high-precision prediction of absolute loading efficiency, but rational prioritization of loading strategies at the decision level. This distinction is critical in the context of extracellular vesicle (EV) systems, which are inherently biologically variable.

Accordingly, Table 1 should be viewed not simply as a collection of loading efficiency values, but as a structured comparison enabling translation of physicochemical diversity into actionable formulation heuristics. The goal is to identify reproducible directional tendencies (e.g., when polarity overrides lipophilicity constraints) that can guide prioritization of loading strategies before experimental screening.

EV membranes differ in lipid composition depending on cellular origin and isolation protocol. Vesicle size distribution, membrane protein content, surface charge density, and experimental loading conditions introduce additional variability. Under such circumstances, achieving high R^2^ values for absolute efficiency prediction may be unrealistic with limited sample sizes.

The moderate continuous regression performance observed (R^2^ between 0.06 and 0.41) must therefore be interpreted in the context of system complexity. Notably, decision-level accuracy remained consistently high across internal (76.5%), predefined external (75%), and repeated random validation (80.5% ± 16.8%). This divergence suggests that descriptor information more robustly encodes relative ranking of loading strategies than absolute efficiency magnitudes.

From a formulation perspective, this ranking-based objective is more relevant. In practical development workflows, the key question is not the exact predicted loading percentage, but which method should be prioritized experimentally.

### 4.2. Mechanistic Interpretation of Descriptor Patterns

The standardized regression coefficients reveal coherent mechanistic trends consistent with known membrane interaction principles.

#### 4.2.1. Passive Incubation

Passive incubation exhibited strong positive association with lipophilicity (LogP) and formal charge. Lipophilicity promotes membrane partitioning into the hydrophobic lipid bilayer, facilitating diffusion-driven incorporation. The negative contribution of PSA and hydrogen bonding descriptors suggests that highly polar molecules exhibit reduced passive partitioning.

This pattern aligns with classical membrane permeability theory, in which hydrophobic compounds preferentially distribute into lipid phases.

The positive coefficient for charge may reflect electrostatic interactions between charged molecules and membrane phospholipid headgroups. However, this effect likely depends on pH and membrane surface potential, which were not explicitly modeled.

#### 4.2.2. Saponin-Mediated Permeabilization

Saponin interacts with membrane cholesterol, transiently increasing permeability. The positive association of hydrogen bonding capacity (HBD, HBA) and PSA suggests enhanced loading of polar molecules under permeabilized conditions.

This finding is mechanistically plausible: membrane disruption reduces reliance on lipophilic partitioning and allows increased incorporation of polar compounds that would otherwise exhibit low passive permeability.

The negative association with charge may reflect differential stability of charged species under permeabilization dynamics.

#### 4.2.3. Electroporation

Electroporation generates transient aqueous nanopores. The observed sensitivity to charge and polarity descriptors is consistent with pore-mediated transport mechanisms. Charged and polar molecules may traverse transient pores more readily than through intact lipid bilayers.

The negative contribution of lipophilicity supports the hypothesis that lipophilic partitioning is less critical under electroporative conditions.

#### 4.2.4. Mechanical Disruption Methods

Freeze–thaw and sonication exhibited broader descriptor sensitivity patterns. Mechanical disruption may reduce dependency on passive diffusion constraints, allowing descriptor contributions to reflect more complex interactions between membrane restructuring and drug physicochemistry.

Sonication displayed relatively higher R^2^ values compared to other methods, suggesting that mechanical disruption may generate more consistent loading patterns under the studied conditions.

From a practical standpoint, these relationships can be condensed into an operational rule-of-thumb for early-stage method selection. Lipophilic, weakly polar molecules tend to be most consistently accommodated by passive incubation due to favorable membrane partitioning. In contrast, highly polar or hydrogen-bond-rich cargos more often benefit from permeability-enhancing strategies such as saponin-mediated loading. Mechanical disruption methods, including freeze–thaw cycling and sonication, provide broader but less descriptor-sensitive applicability and may serve as general fallback strategies when no clear diffusion- or permeabilization-driven preference emerges. These trends are not deterministic predictions but are intended to rationally narrow the experimental search space prior to empirical optimization.

### 4.3. Ampicillin Misclassification as a Mechanistic Case Study

Ampicillin was misclassified during predefined external validation. Experimentally, saponin-mediated loading yielded maximal efficiency, while the model predicted passive incubation. Ampicillin is characterized by high polarity, multiple hydrogen bonding sites, and negative charge. While permeabilization mechanisms theoretically favor such compounds, the model’s linear structure may not fully capture nonlinear threshold effects associated with cholesterol-mediated membrane disruption.

Additionally, formal charge representation may not sufficiently describe pH-dependent ionization dynamics. Ionization state-adjusted descriptors (e.g., fraction ionized at loading pH) could potentially improve classification accuracy in future work.

The single misclassification does not indicate systematic bias, as repeated random validation maintained stable performance. Rather than undermining the validity of the framework, the ampicillin case underscores its role as a decision-support tool that must be applied with awareness of its applicability domain and mechanistic assumptions. In prospective use, misclassification-prone regions of descriptor space, such as highly polar, multi-ionizable compounds, could be flagged a priori, prompting formulators to treat model recommendations as hypothesis-generating rather than deterministically prescriptive. Future work could explicitly target such chemotypes by (i) enriching the training set with additional polar, ionizable compounds; (ii) augmenting the descriptor set with pH-adjusted, microenvironment-sensitive ionization descriptors; and (iii) exploring local nonlinear models or hybrid physics-informed/ML approaches that better accommodate permeabilization-driven transport phenomena.

### 4.4. Robustness and Bias–Variance Considerations

Given the limited dataset size (n = 21), overfitting represents a substantial risk. Elastic Net regularization was selected to balance bias and variance under potential descriptor collinearity.

Repeated random external validation demonstrated that performance did not collapse under resampling. The observed variability (±16.8%) is consistent with the statistical uncertainty expected in small experimental datasets and does not indicate structural instability of the descriptor-based framework.

Nevertheless, confidence intervals around decision accuracy would likely narrow with larger datasets. Future expansion of compound diversity remains essential for improving predictive robustness.

### 4.5. Applicability Domain and Predictive Boundaries

Incorporation of leverage-based applicability domain analysis enhances translational reliability. The calculated threshold (h* = 1.143) provides an explicit criterion for identifying extrapolation risk.

Descriptor-based models are inherently limited to the chemical space represented in training data. Prospective predictions for compounds exceeding leverage thresholds should be interpreted cautiously and ideally validated experimentally.

Integration of applicability domain assessment distinguishes the present framework from purely empirical or black-box approaches.

### 4.6. Comparison with Empirical Screening Approaches

Conventional EV loading strategy selection frequently relies on empirical parallel screening of multiple techniques. While effective, this approach increases resource consumption and experimental variability.

The present framework does not eliminate the need for experimental validation. Rather, it reduces the search space by rationally prioritizing likely optimal methods.

Such integration of descriptor-informed modeling into EV formulation workflows represents a conceptual advancement toward more systematic development strategies. In a typical EV formulation project, the present framework can be used to pre-rank routinely available loading methods for a new small-molecule candidate and restrict experimental evaluation to the top one or two predicted strategies, instead of testing the full panel of five techniques. Even at an accuracy level of approximately 75–80%, such descriptor-guided triage has the potential to meaningfully reduce consumable use and EV material demand, especially for scarce or patient-derived vesicle preparations.

### 4.7. Limitations

Several limitations must be acknowledged. First, the dataset size (n = 21) constrains statistical resolution and descriptor-space coverage. Second, the descriptor set is restricted to basic physicochemical properties and does not incorporate EV lipidomic or proteomic characteristics. Third, the framework relies on linear modeling assumptions and does not explicitly account for dynamic membrane interaction parameters. Finally, variability in experimental loading conditions may introduce additional sources of uncertainty. These limitations constrain both mechanistic resolution and predictive precision.

This study has three main limitations. First, the dataset comprises 21 small-molecule cargos characterized within a single EV system and protocol framework, which constrains the chemical and biological diversity that the current models can reliably capture. Second, the descriptor set is restricted to basic physicochemical properties of the cargos and does not yet incorporate EV membrane characteristics or protocol-level variables, so the framework cannot address inter-EV or inter-laboratory variability explicitly. Third, the evaluation relies on retrospective data; although we used multiple complementary validation schemes, we did not perform a prospective test where model-guided method selection is experimentally verified for new compounds. These constraints delimit the present framework to decision-support within descriptor space similar to the training set and underscore the need for cautious interpretation when extrapolating to markedly different cargos or EV systems. An additional limitation arises from the biological heterogeneity of extracellular vesicles. EVs derived from different cellular or microbial sources may exhibit substantial variation in membrane lipid composition, protein content, surface charge, and structural organization. For example, vesicles originating from Gram-negative and Gram-positive bacteria differ fundamentally in envelope architecture, which can alter drug–membrane interactions and loading behavior. Consequently, the descriptor–response relationships identified in the present study should be interpreted as applicable to EV systems of comparable physicochemical characteristics rather than universally transferable across all vesicle types. Future work should systematically evaluate framework performance across EVs of diverse biological origin and membrane composition to establish the boundaries of generalizability.

### 4.8. Future Directions

Future investigations should focus on expanding the chemical diversity of the dataset to further improve descriptor-space coverage and statistical robustness. Incorporation of ionization-state–adjusted descriptors and EV membrane–specific parameters, such as lipid composition or surface-associated biomolecules, may allow more precise representation of drug–membrane interactions [19]. In addition, exploring nonlinear or hybrid modeling approaches could help capture transport phenomena that are not fully described by linear relationships. Systematic evaluation of inter-batch and inter-source reproducibility will also be essential to determine how broadly the framework can be applied across different EV preparations. Collectively, such developments would strengthen both the mechanistic resolution and the translational utility of descriptor-guided loading strategy selection [19,20].

## 5. Conclusions

This study introduces a descriptor-based, mechanistically interpretable decision-support framework for selecting extracellular vesicle loading strategies for small-molecule cargos within EV-based drug delivery development. By focusing explicitly on ranking-based prioritization among five routinely used loading methods, rather than on high-precision prediction of absolute loading efficiencies, the framework aligns with the practical needs and biological heterogeneity of EV-based formulation workflows.

Using a chemically diverse 21-compound dataset anchored in experimentally measured loading efficiencies, we developed separate Elastic Net models for passive incubation, electroporation, saponin-mediated permeabilization, freeze–thaw cycling, and sonication, and showed that, despite modest continuous regression performance, decision-level accuracy for the optimal method consistently approached or exceeded 75% across complementary validation schemes. These results demonstrate that a compact, mechanistically motivated descriptor set contains sufficient information to support robust triage among standard EV loading strategies, thereby offering a rational alternative to purely empirical, trial-and-error screening in early-stage formulation design.

At the same time, misclassification analysis and applicability-domain assessment delineate clear boundaries for the current models and identify compound classes, such as highly polar, multi-ionizable drugs, for which additional data, refined ionization descriptors, or nonlinear modeling may be required. Future work integrating EV membrane and protocol-level descriptors, expanding the chemical and biological space, and prospectively testing model-guided method selection in new EV systems will be essential to fully exploit descriptor-guided loading strategy selection across the breadth of EV-based nanocarrier platforms.

Overall, the present work provides an experimentally grounded, quantitatively defined starting point for incorporating molecular descriptor-based decision-support into the rational design of EV-based dosage forms and controlled-release strategies, with the potential to streamline early-stage EV formulation workflows and to prioritize experimental resources in drug delivery development programs.

## Figures and Tables

**Table 1:** Drug Dataset with FDA-Defined BCS Classification and Physicochemical Descriptors. (Drugs highlighted in grey indicate the external test set used for independent validation across distinct BCS classes and physicochemical profiles).

Drug Name	BCS Class	LogP	MW (g/mol)	Solubility (μg/mL)	HBD	HBA	PSA (Ų)	Charge	Passive Incubation (%)	Electroporation (%)	Saponin (%)	Freeze–Thaw (%)	Sonication (%)
Doxorubicin	NC (IV-only)	1.27	543.5	50	4	8	124	+1	72	8	18	15	4
Paclitaxel	NC (no FDA oral IR)	3.97	853.9	0.3	2	11	97	0	75	2	25	12	8
Cisplatin	NC (IV-only)	−2.50	300.0	3.5	0	2	0	0	65	7	12	18	6
Gemcitabine	NC (IV-only)	−1.20	263.2	100	3	5	95	0	68	9	22	22	7
Docetaxel	NC (IV-only)	4.10	861.9	0.1	2	11	98	0	77	1.5	28	10	6
Methotrexate	IV	−1.85	454.4	0.3	4	9	168	−2	5	52	68	32	45
Riboflavin	III	−0.60	376.4	1.2	5	8	149	0	8	48	71	38	48
Ampicillin	III	0.87	349.4	1.0	3	8	115	−1	3	55	74	35	51
Furosemide	IV	2.03	330.7	0.5	2	5	99	−1	70	5	20	8	15
Warfarin	II	2.92	308.3	0.14	1	4	49	0	72	3	18	9	11
Digoxin	NC (conflicting II/III; NTI)	1.26	780.9	0.05	5	12	206	0	65	18	35	40	38
Chloroquine	I	3.81	319.9	0.7	2	4	47	+1	68	8	22	14	18
Irinotecan	NC (primarily IV; insufficient FDA BCS)	3.27	586.7	0.2	2	9	106	0	71	5	24	11	13
5-Fluorouracil	III/NC (limited oral IR context)	−0.89	130.1	12.2	2	3	66	0	55	10	15	19	9
Caffeine	I	0.16	194.2	21.5	0	3	58	0	52	11	14	21	11
Quercetin	NC (not approved as drug)	1.83	302.2	0.003	5	7	131	0	69	4	19	12	14
Resveratrol	NC (not approved as drug)	3.05	228.2	0.3	3	3	60	0	73	6	21	13	16
Sildenafil	II	2.71	474.6	3.5	2	6	87	0	74	4	20	10	12
Curcumin	NC (not approved as drug)	3.97	368.4	0.003	2	4	93	0	76	2	26	9	7
Pirarubicin	NC (IV-only)	1.34	557.5	45	4	8	126	+1	70	9	19	16	5
Tamoxifen	II	4.30	371.4	0.01	1	2	39	0	79	1	15	7	5

Note: BCS = Biopharmaceutics Classification System (assigned according to FDA/ICH M9 definitions based on solubility and permeability criteria); NC = not classifiable under FDA BCS criteria (e.g., IV-only drug, no FDA-approved immediate-release oral product, or insufficient/conflicting public data); LogP = octanol–water partition coefficient (lipophilicity); MW = molecular weight (g/mol); HBDs = hydrogen bond donors; HBAs = hydrogen bond acceptors; PSA = polar surface area (Å^2^); Charge = net molecular charge at physiological pH (7.4); NTI = Narrow Therapeutic Index drug; Passive Incubation, Electroporation, Saponin, Freeze–Thaw, and Sonication (%) indicate experimentally reported extracellular vesicle (EV) drug loading efficiencies for the respective loading methods.

**Table 2:** Internal LOOCV Performance (Training Set, n = 17).

Loading Method	MAE (%)	RMSE (%)	R^2^ (LOOCV)
Passive Incubation	13.70	20.62	0.06
Electroporation	9.32	13.76	0.12
Saponin	10.56	15.45	0.12
Freeze–Thaw	5.90	8.09	0.31
Sonication	8.64	10.57	0.41

R^2^ values ranged between 0.06 and 0.41, reflecting moderate continuous regression performance. Mechanical methods (freeze–thaw and sonication) exhibited comparatively higher explained variance.

**Table 3:** External Validation Results.

Compound	True Optimal Method	Predicted Optimal Method
Ampicillin	Saponin	Passive
Furosemide	Passive	Passive
Caffeine	Passive	Passive
Sildenafil	Passive	Passive

## Data Availability

The decision-support framework (Excel-based predictor and Python scripts for model training/validation) is openly available on GitHub at: https://github.com/chege54/ev-loading-decision-tool/tree/v1.0.0 (accessed on 6 March 2026)).

## Supplementary Materials

The following supporting information can be downloaded at: https://www.mdpi.com/article/10.3390/pharmaceutics18030384/s1. Table S1. A Descriptor-Based Decision Tool for Extracellular Vesicle Loading Strategy Selection.
